# Anti-tumor effect of avadomide in gemcitabine-resistant pancreatic ductal adenocarcinoma

**DOI:** 10.1007/s00280-023-04531-w

**Published:** 2023-07-26

**Authors:** Hidemi Nishi, Kunihito Gotoh, Yoshito Tomimaru, Shogo Kobayashi, Kazuki Sasaki, Yoshifumi Iwagami, Daisaku Yamada, Hirofumi Akita, Tadafumi Asaoka, Takehiro Noda, Hidenori Takahashi, Masahiro Tanemura, Yuichiro Doki, Hidetoshi Eguchi

**Affiliations:** Department of Gastroenterological Surgery, Graduate School of Medicine, Osaka University, Suita, Japan

**Keywords:** Avadomide, Gemcitabine-resistant, Pancreatic cancer, Xenograft murine model

## Abstract

**Purpose:**

Although gemcitabine-based chemotherapy is most recommended for pancreatic ductal adenocarcinoma (PDAC), its effectiveness is limited because of drug resistance. Given thalidomide’s anti-tumor effects in solid tumors, we investigated the effect of avadomide, a novel thalidomide analog, on PDAC and explored its anti-tumor mechanisms.

**Methods:**

PDAC cell lines, including gemcitabine-resistant (GR) clones derived from MiaPaCa2 cells, were used to evaluate the effects of avadomide. An annexin V assay, a cell cycle assay, and western blot analysis were performed to explain the mechanism of avadomide as an anti-tumor reagent. Moreover, we investigated the anti-tumor effect on tumor growth using a subcutaneous xenograft murine model.

**Results:**

Avadomide showed anti-tumor effects in human PDAC cell lines. The proportion of apoptotic cells and G0/G1 phase cells after avadomide treatment increased, especially in the GR PDAC clones. Western blot analysis also showed the induction of the apoptotic pathway by inhibiting the NF-κB process and G1 phase cell cycle arrest. The xenograft murine model revealed that the proportion of viable cells in the avadomide-treated group was lower than that in the untreated group.

**Conclusion:**

Our findings suggest that avadomide could be a novel therapeutic option to overcome gemcitabine resistance in patients with PDAC.

**Supplementary Information:**

The online version contains supplementary material available at 10.1007/s00280-023-04531-w.

## Introduction

The incidence and mortality rates of pancreatic ductal adenocarcinoma (PDAC) have increased gradually, particularly in developed countries [1]. Although the percentage has doubled in this decade from 5 to 10%, the 5-year survival rate of PDAC remains low [2, 3]. A few reasons for poor prognosis are as follows: only approximately 20% of patients undergo curative resection since PDAC is difficult to diagnose in the early stages [4], more than 80% of patients experienced recurrence even after curative resection [5], and few chemotherapies are known to be effective for PDAC, except gemcitabine, which is at the heart of chemotherapy despite growing resistance with more than 50 identified mechanisms [6]. New molecular therapies for PDAC have been developed over the years [7].

Thalidomide and its analogs, lenalidomide and pomalidomide, have been proven to exhibit anti-tumor effects, and are used as therapeutic agents against hematological malignancies [8]. Despite its well-known anti-tumor effects, only a few studies have reported its effects against solid cancers [9, 10]. Lenalidomide and pomalidomide have shown anti-tumor effects against PDAC in vitro and in vivo [7,11]; however, clinical trials using these drugs could not prove their efficacies [12, 13].

In recent years, avadomide, a newly developed thalidomide analog, has been reported to have stronger anti-tumor effects against some hematological malignancies than the current standard therapy, which is based on thalidomide and its analogs. For example, avadomide shows a stronger anti-tumor effect against diffuse large B-cell lymphoma than lenalidomide [14]. In addition, avadomide has been shown to inhibit tumor growth in hepatocellular carcinoma [15]. Some clinical trials have also shown its safety and efficacy for non-Hodgkin lymphoma [16, 17]. However, little is known about its anti-tumor mechanism and effect on PDAC.

In this study, we aimed to evaluate the therapeutic effect of avadomide using the human PDAC cell with a focus on two GR clones in vivo and in vitro*,* particularly with regard to pharmacological effects.

## Materials and methods

### Cell lines and cell culture

The human PDAC cell lines Panc1, BxPC3, PSN1, and MiaPaCa2 were purchased from the Japan Cancer Research Resources Bank (Tokyo, Japan). These cell lines were maintained in Dulbecco’s modified Eagle’s medium (DMEM) supplemented with 10% fetal bovine serum (FBS), penicillin (100 U/mL), and streptomycin (100 U/mL) at 37 °C in a humidified incubator with 5% CO_2_.

### Reagents

Avadomide (Med Chem Express, NJ, USA) was dissolved in dimethyl sulfoxide (DMSO; 100 mM), and gemcitabine (GEM; Eli Lilly Pharmaceuticals, IN, USA) was dissolved in diluted water (1 mg/mL) and stored at − 80 °C until further use.

### Establishment of gemcitabine-resistant cell clones

GR cells were generated by exposure to gemcitabine for 2 months, as described previously [18]. Parent MiaPaCa2 (MiaPaCa2-P) cells were exposed to a concentration of 1 ng/mL gemcitabine solution. When MiaPaCa2-P showed tolerance to the gemcitabine solution, gemcitabine concentration was gradually increased. The final concentration of gemcitabine solution was 20 ng/mL. Two GR cell clones (MiaPaCa2-GRs: MiaPaCa2-GR1 and MiaPaCa2-GR2) were derived from MiaPaCa2-P.

### Growth inhibition assay

Growth inhibition was assessed using 3- (4-,5-dimethylthiazol-2-yl)-2,5-diphenyl tetrazolium bromide (MTT) (Sigma-Aldrich Co., MO, USA), as described previously [19]. In brief, each cell line was incubated for 72 h with various concentrations of avadomide (15.6, 31.2, 62.5, 125, 250, and 500 μM), gemcitabine (3.13, 6.25, 12.5, 25, and 50 ng/ml), or combinations of both. After re-incubation for 4 h with the MTT solution, an acid-isopropanol mixture was added to dissolve the resulting formazan crystals. The absorbance was measured using a microplate reader at a wavelength of 550 nm with a 650 nm reference, and the results were expressed as a percentage of absorbance relative to that of the untreated controls.

### Flow cytometric analysis

Flow cytometry was performed using a BD FACS Canto II (BD Biosciences, NJ, USA). Doublet cells were eliminated using FSC-A (forward scatter-area)/FSC-H (forward scatter-height) and SSC-A (slide scatter-area)/SSC-H (slide scatter-height). Approximately 10,000 cells were tested for each sample measuring Annexin V assay and DNA cell cycle assay. Flow cytometric analyses were performed using Flow Jo version 10.7.2 (Digital Biology Co. Ltd., Tokyo, Japan).

### Annexin V assay

The binding of annexin V was used as a sensitive method for measuring early apoptosis, as described previously [20]. PDAC cells were exposed to avadomide (200 μM) or gemcitabine (Panc1, BxPC3, PSN1, and MiaPaCa2-P: 10 ng/mL, MiaPaCa2-GRs: 50 ng/mL) for 36 h. The collected cells were treated with Annexin V-FITC Apoptosis Detection Kits (K101-400, Bio Vision Research Products, CA, USA) according to the manufacturer’s protocol. Collected cells were resuspended in 500 μL of binding buffer and incubated with 5 μL of annexin V-FITC and 5 μL of propidium iodide at room temperature for 5 min in the dark. After incubation, cells were analyzed by flow cytometry.

### DNA cell cycle analysis

The cell cycle was assessed as previously described [21]. Before treatment, PDAC cells were starved in DMEM lacking FBS for 24 h, and then exposed to avadomide (200 μM) for 30 h. Collected cells were fixed in 70% ethanol in ice for 4 h and treated with 0.1 mg/mL RNase A (QIAGEN, Venlo, Netherlands) at 37 °C for 30 min in the dark. Cells were incubated with 50 mg/mL PI solution (Immunostep, Salamance, Spain) for 5 min in the dark and were analyzed by flow cytometry.

### Western blot analysis

Western blotting (WB) was performed as described previously [22]. Briefly, total protein was extracted from the cells. Aliquots of total protein (10 μg) were electrophoresed on sodium dodecyl sulfate–polyacrylamide and 12% Tris–HCL gels (Bio-Rad Laboratories, CA, USA). The separated proteins were transferred onto polyvinylidene difluoride membranes (Bio-Rad Laboratories) and incubated with primary antibodies overnight at 4 °C. The membranes were probed with the following antibodies: cyclin D1, p27 Kip1, CDK2, p18 INK 4C, CDK6, cyclin D3, p21 Waf1/Cip1, CDK4, pIκBα, IκBα, pro caspase 3, Ikaros, CK2α, c-Myc (1:1000 dilution; Cell Signaling Technology, Inc., MA, USA), and anti-β actin (1:3000 dilution; Sigma, Tokyo, Japan). Bound primary antibodies were detected using secondary antibodies (Cell Signaling Technology, Inc.) in a 1:10,000 dilution for anti-β actin and 1:2000 dilution for the other antibodies.

### Animal and in vivo allograft experimental models

The animal experimental protocol was approved by the Animal Experiments Committee at Osaka University (no.01-058-002) and was conducted according to the National Institutes of Health guidelines for the use of experimental animals. Six-week-old male nude mice (BALBc nu/nu) were purchased from CLEA Japan Inc. (Tokyo, Japan) and housed under specific pathogen-free conditions in a biological cabinet at the Laboratory Animal Facility of Osaka University School of Medicine. We established a PDAC xenograft model in mice by injecting 1 × 10^7^ cells suspended in 100 μL of phosphate buffer saline (PBS) into the subcutaneous tissue. Subsequently, 4 weeks after injection, the mice were divided into two groups: avadomide (0.5 mg/kg) (avadomide group) or DMSO equivalent to avadomide dilution (non-treatment group), with oral administration five times a week. Four weeks after treatment, the mice were sacrificed and subcutaneous PDAC tumors were extracted and fixed in 10% formalin solution.

### Hematoxylin and eosin staining

Hematoxylin and eosin staining was performed automatically using a Tissue-Tek® DRS (Sakura Finetek, Tokyo, Japan). The stained tissues being 3.5 μm-thick were observed under a microscope and analyzed using a BR-X analyzer (Keyence Corporation, Osaka, Japan) to discriminate viable from necrotic tissue areas. The percentage of viable tissue area was calculated as the difference in color.

### Statistical analysis

Continuous variables were expressed as mean ± standard deviation (S.D.) and compared using Student’s *t*-test. A *P* value < 0.05 denoted the presence of a statistically significant difference. Statistical analysis was performed using JMP software version 16.0.0 (SAS Institute Inc., NC, USA).

### Combination index

The combination index (CI) was used to analyze the combined drug effect using a previously described method [23]. The CI was calculated according to the following formula: $CI= (Da/D30a) + (Db/D30b)$, where D_30_a and D_30_b are the concentrations of drug A/B required to produce 30% of the effect.

## Results

### Effect on PDAC cells

**Fig. 1:**
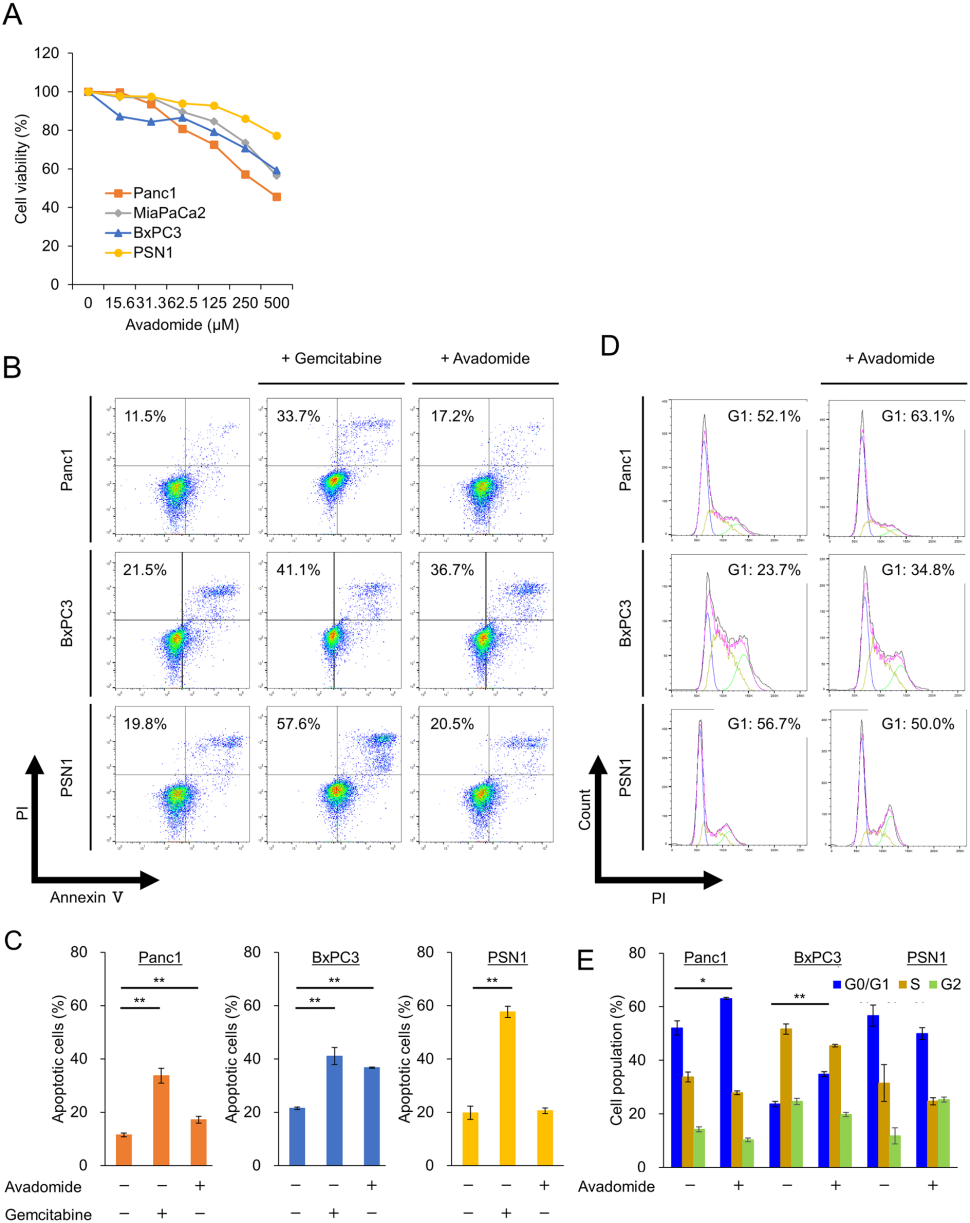
Effect of avadomide on PDAC cells in the apoptosis and cell cycle phases, **A** MTT assay showing the anti-tumor effects of avadomide in four PDAC cell lines. Data are represented as mean of triplicate independent experiments. **B** Representative dot plots from flow cytometry analysis illustrating apoptotic status by Annexin V assay. Panc1, BcPX3, and PSN1 after treatment with placebo, 10 ng/mL gemcitabine, and 200 μM avadomide for 36 h, respectively. **C** Statistical analysis of proportion of apoptotic cells after gemcitabine and avadomide therapy compared with placebo. Bar graphs indicate the percentage of apoptotic cells. Data are represented as mean ± S.D. of at least triplicate samples. Similar results were obtained from three independent experiments. *p < 0.05, **p < 0.01, compared with control. **D** Representative quantitative plots from flow cytometry analysis illustrating cell cycle status by DNA cell cycle assay. Panc1, BxPC3, and PSN1 incubated with or without 200 μM avadomide for 36 h. Approximately 10,000 cells were tested for each sample. **E** Statistical analysis of proportion of cell cycle status after avadomide therapy. Bar graphs indicate the percentage of each cell cycle status proportion. Each bar represents the mean ± S.D. of at least triplicate samples. Similar results were obtained from three independent experiments. *p < 0.05, **p < 0.01, compared with control

The growth inhibitory effect of avadomide was observed in the four PDAC cell lines (Fig. 1A). To investigate the mechanism underlying the effect of avadomide on PDAC cell lines, we performed an annexin V assay and cell cycle assay. Avadomide induced apoptosis (Figs. 1B, C, 3A, B) and increased the proportion of G0/G1 phase cells (Figs. 1D, E, 4A, B) in Panc1, BxPC3, and MiaPaCa2 cells.

### Establishment of GR PDAC clones

We established two independent gemcitabine-resistant MiaPaCa2 clones (MiaPaCa2-GR1 and MiaPaCa2-GR2). Compared to MiaPaCa2-P, MiaPaCa2-GRs showed faster growth curves, particularly the MiaPaCa2-GR2 clone (Online resource 1, Supplementary Fig. 1A). We confirmed MiaPaCa2-GRs were significantly resistant to gemcitabine (**p < 0.01) (Online resource 1, Supplementary Fig. 1B).

### Antiproliferative effect of avadomide in MiaPaCa2-GRs

**Fig. 2:**
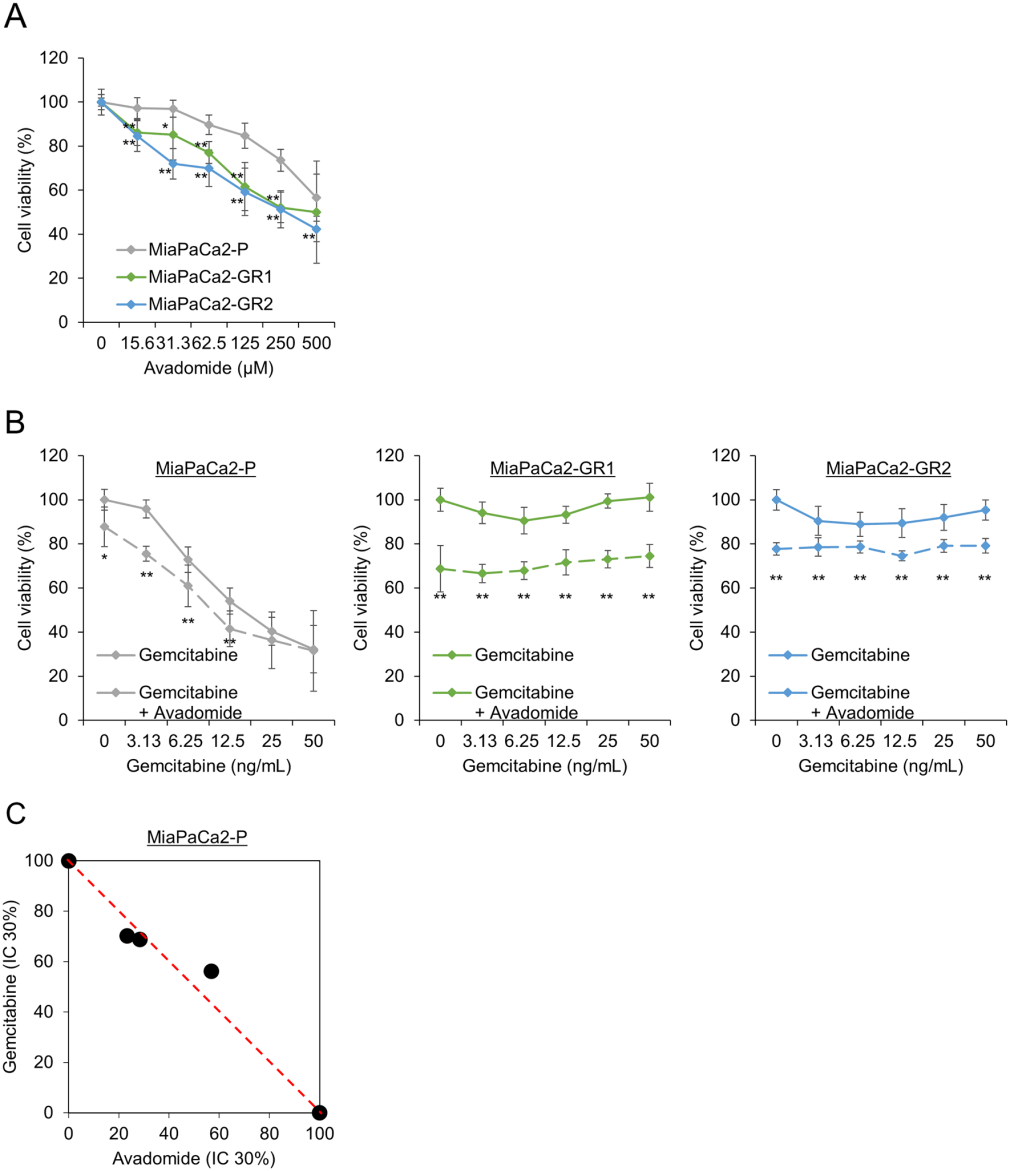
Characteristics of gemcitabine-resistant MiaPaCa2 cell clones, **A** MTT assay confirmed the anti-tumor effect of avadomide in MiaPaCa2-GR clones. **B** MTT assay showed an additive effect on MiaPaCa2-P cells from combination therapy. Data are represented as mean ± S.D. of triplicate independent experiments. *p < 0.05, **p < 0.01, compared with control. **C** Normalized isobologram analysis. The diagonal red dotted line indicates additivity, and the black symbol shows dose requirements to achieve 30% cancer cell inhibition. Data points below the dotted line indicate synergism, and data points above the dotted line indicate antagonism

The growth inhibitory effect of avadomide was also observed in the MiaPaCa2-GRs and was stronger in these clones than in MiaPaCa2-P (Fig. 2A). The efficacy of combination therapy of avadomide with gemcitabine was observed in MiaPaCa2-P (Fig. 2B). The CI showed an additive effect in MiaPaCa2-P (Fig. 2C).

### Avadomide induces apoptosis in MiaPaCa2-GRs

**Fig. 3:**
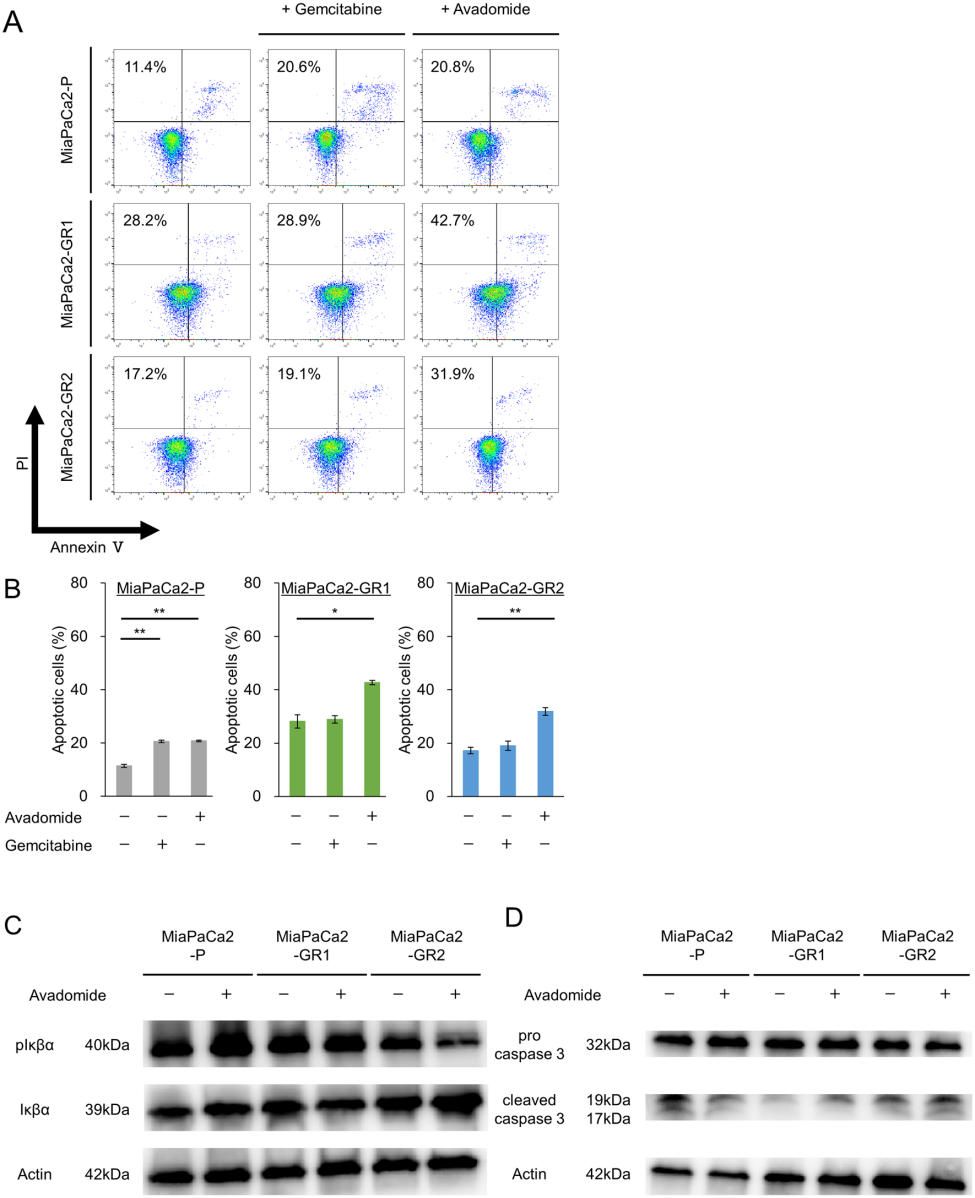
Effect of avadomide on apoptosis induction in PDAC cells and expression of NF-κB pathway proteins, **A** Representative dot plots from flow cytometry analysis illustrating apoptotic status by Annexin V assay. MiaPaCa2-P and MiaPaCa2-GR clones, after treatment with placebo, 10 ng/mL (MiaPaCa2-P) or 50 ng/mL (MiaPaCa2-GR clones) of gemcitabine, 200 μM of avadomide for 36 h, respectively. **B** Statistical analysis of proportion of apoptotic cells after gemcitabine and avadomide therapy compared with placebo. Bar graphs indicate the percentage of apoptotic cells. Data are represented as mean ± S.D. of at least triplicate samples. Similar results were obtained from three independent experiments. *p < 0.05, **p < 0.01, compared with control. **C** The effect of avadomide on the protein levels of pIκBα and IκBα in PDAC cells. **D** The effect of avadomide on the protein levels of cleaved caspase 3

To investigate the mechanism underlying the effect of avadomide on MiaPaCa2-P and MiaPaCa2-GRs, we performed an annexin V assay. Although gemcitabine induced apoptosis only in MiaPaCa2-P cells, avadomide induced apoptosis in MiaPaCa2-P cells and MiaPaCa2-GRs (Fig. 3A, B). To confirm the details of the apoptosis induction, we examined the role of avadomide in the NF-κB process, which increases the expression of proliferative factors. NF-κB is inactivated by forming a complex with IκBα until the proliferation signal stimulates the IKK complexes. Phosphorylation of IκBα induced by IKK complexes causes NF-κB release and activates NF-κB. We evaluated whether the expression of phosphorylated IκBα, which is an upstream molecule of the NF-κB process, was decreased by avadomide in MiaPaCa2-GRs (Fig. 3C). Finally, avadomide was shown to elevate cleaved caspase 3 in the MiaPaCa2-GRs (Fig. 3D).

### Avadomide induces G1 phase cell cycle arrest in MiaPaCa2-GRs

**Fig. 4:**
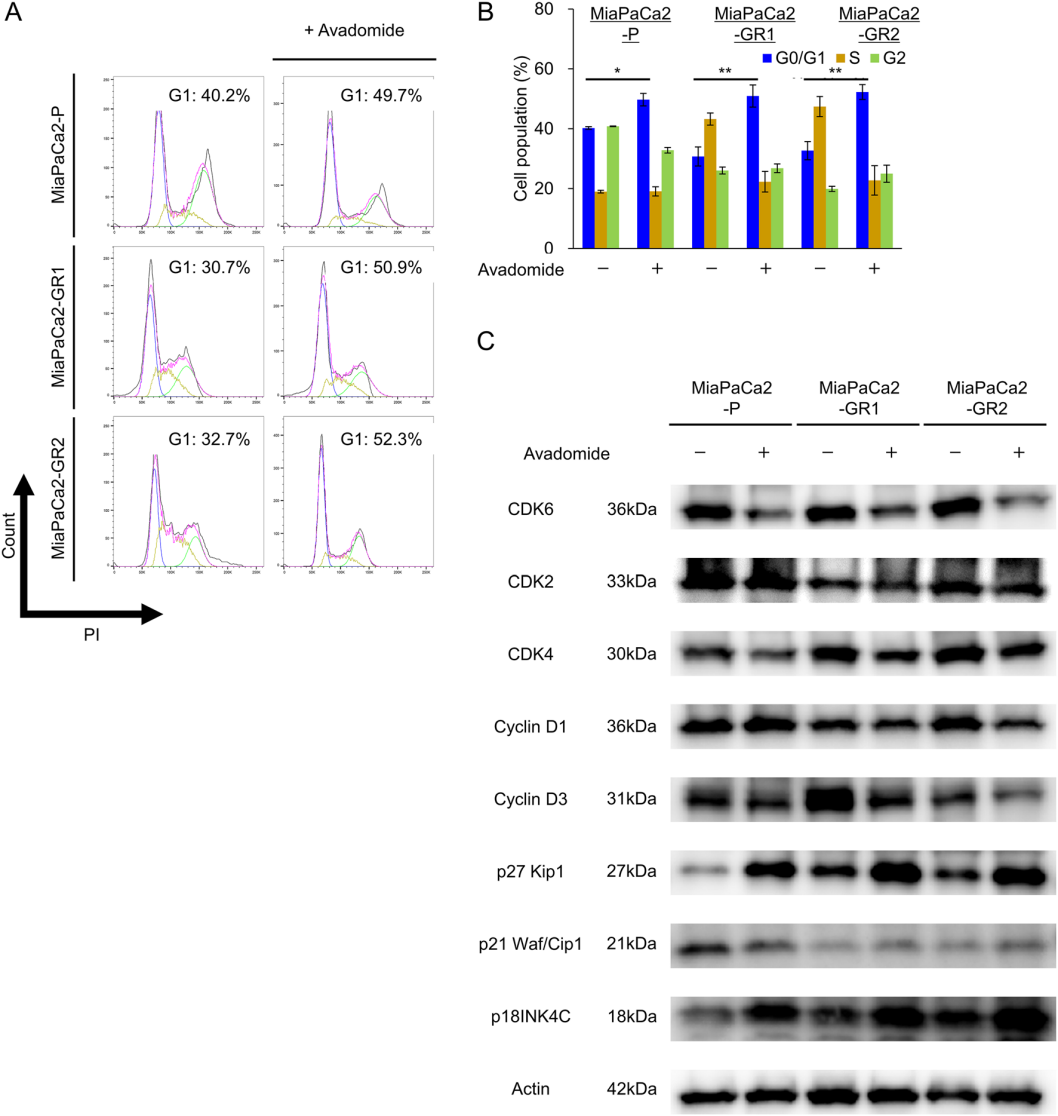
Effect of avadomide on cell cycle distribution in PDAC cells, **A** Representative quantitative plots from flow cytometry analysis illustrating cell cycle status by DNA cell cycle assay. MiaPaCa2-P and MiaPaCa2-GR clones incubated with or without 200 μM of avadomide for 36 h. Approximately 10,000 cells were tested for each sample. **B** Statistical analysis of proportion of cell cycle status after avadomide therapy. Bar graphs indicate the percentage of each cell cycle status proportion. Each bar represents the mean ± S.D. of at least triplicate samples. Similar results were obtained from three independent experiments. *p < 0.05, **p < 0.01, compared with control. **C** The effect of avadomide on the protein levels of CDK4/6, CDK2, CyclinD1/3, p18INK4C, p21Waf/Cip1, and p27Kip1

To investigate the mechanism underlying the effect of avadomide on MiaPaCa2-P and MiaPaCa2-GRs, we performed a cell cycle assay. Avadomide increased the proportion of G0/G1 cells in MiaPaCa2-P cells and MiaPaCa2-GRs (Fig. 4A, B). To confirm the details of the cell cycle, we evaluated proteins related to the G1/S checkpoint. MiaPaCa2-P and MiaPaCa2-GRs showed elevated expression of p18INK4C, which is a specific inhibitor of the G1 phase. The expression of p21 Waf/Cip and p27 Kip, a non-specific inhibitor of cyclin-dependent kinases (CDKs), was also elevated. CDK4/6 and cyclin D1/3, which play important roles in the G1/S checkpoint, decreased after avadomide treatment (Fig. 4C). CDK2 level, which aids in replicative DNA synthesis in the S phase, remained unchanged. Briefly, avadomide increased CDK inhibitor expression and caused G1/S checkpoint inhibition.

### Avadomide modulates the expression of Ikaros and c-Myc in MiaPaCa2-GRs

Avadomide has demonstrated antiproliferative activity with hematological malignancy, especially in diffuse large B-cell lymphoma (DLBCL) cell lines, through proteasomal degradation of Ikaros [14]. Ikaros is a zinc finger transcriptional regulator mainly related to hematopoiesis, originally thought to be a tumor suppressor, although several reports showed that high expression of Ikaros may be associated with the poor outcome of solid cancers [24, 25].

**Fig. 5:**
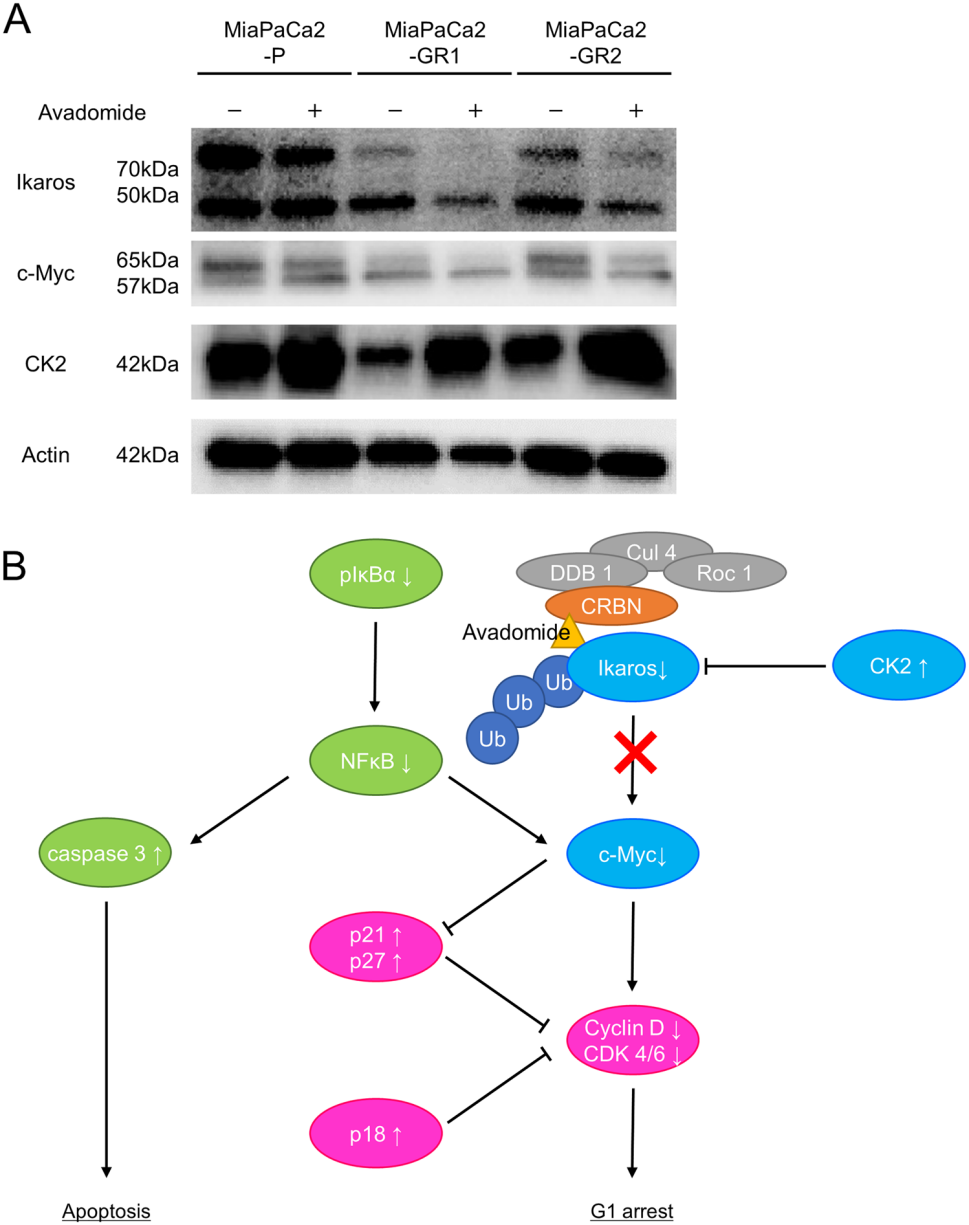
Avadomide-modulated expression of Ikaros occurs in PDAC cells followed by downregulation of c-Myc as with hematological malignancies, **A** The effect of avadomide on the protein levels of Ikaros, c-Myc, and CK2α. **B** Estimated working model; we show that avadomide induced proteasomal degradation of Ikaros in PDAC cells, followed by c-Myc upregulation as with hematological malignancies. Elevation of CK2, which controls Ikaros, was possibly caused by negative feedback of Ikaros degradation

To investigate the mechanism underlying the antiproliferative effect of avadomide on MiaPaCa2-P and MiaPaCa2-GRs, we evaluated proteins related to the expression of Ikaros. The expression of Ikaros was decreased, and that of c-Myc, a transcriptional factor that plays a critical role in tumorigenesis and is suppressively modulated by Ikaros, was also decreased (Fig. 5A). Moreover, the expression of CK2 (casein kinase II), which contributes to tumor maintenance of pancreatic cancer and poor prognosis in solid cancers [26], and suppressively regulates Ikaros function [27], was elevated by a possible negative feedback mechanism (Fig. 5A).

Avadomide thus directly decreased Ikaros expression, resulting in decreased c-Myc expression, subsequently inhibiting proliferation of MiaPaCa2-P and MiaPaCa2-GRs through several pathways (Fig. 5B).

### Avadomide enhances anti-tumor effects in a xenograft mouse model

**Fig. 6:**
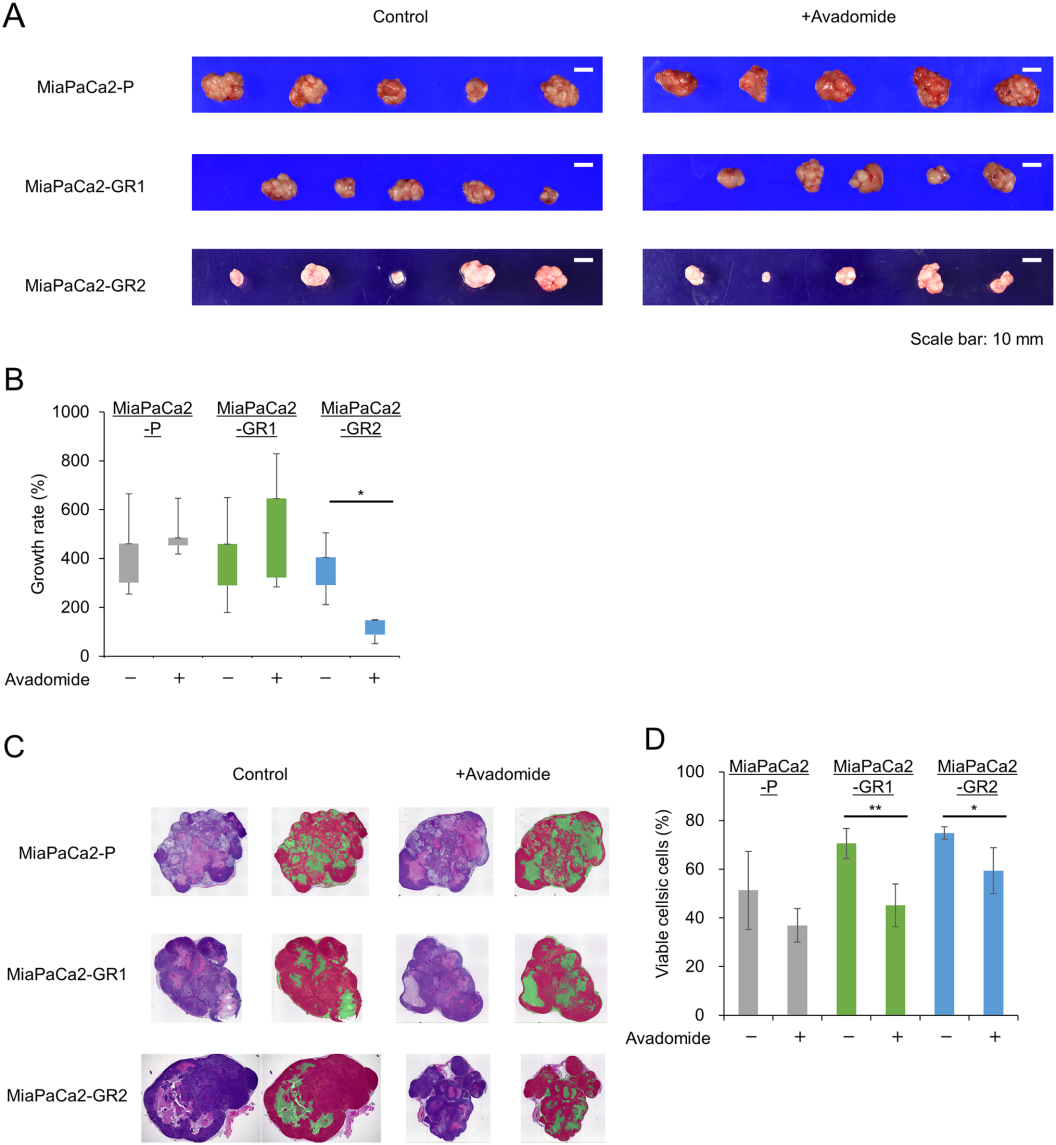
Effect on subcutaneous tumors, **A** Tumor removed from subcutaneous area. **B** Tumor growth rate differed between cell lines. MiaPaCa2-GR2 was the most suppressive. **C** Hematoxylin and eosin staining of the tumor tissues. **D** The proportion of viable cells was low in all cell lines. Bar graphs indicate the percentage of viable cells of each resected specimen. Each bar represents the mean ± S.D. of all samples. *p < 0.05, **p < 0.01 compared with control

To assess the anti-tumor activity of avadomide in vivo, nude mice bearing xenografts were randomly divided into two groups (control and avadomide). The tumor weights in the two groups were 1.89 [1.11–2.09] g vs. 2.62 [2.56–2.84] g in MiaPaCa2-P, 0.72 [0.54–1.09] vs. 0.94 [0.74–1.09] g in MiaPaCa2-GR1, and 0.29 [0.29–0.42] vs. 0.79 [0.13–0.80] g in MiaPaCa2-GR2, respectively. Although the growth rate was significantly suppressed in the MiaPaCa2-GR2 group, tumor weight was not significantly different between the two groups (Fig. 6B).

Furthermore, evaluation of resected specimens stained with hematoxylin and eosin showed that the proportion of viable tissue area decreased in all cell lines, especially in MiaPaCa2-GRs (*P* = 0.01 and *P* = 0.05, respectively for GR1 and GR2) (Fig. 6C, D).

## Discussion

In the present study, avadomide showed anti-tumor effects on PDAC cells in vitro and in vivo, particularly on GR PDAC clones, via repression of transcription through NF-κB process inhibition and cell cycle arrest by increased expression of CDK inhibitors.

Avadomide is expected to be a new therapeutic option for refractory DLBCL patients, and its safety and efficacy for DLBCL have been examined [28]. Because first-line chemotherapies for PDAC are limited, gemcitabine combined with albumin-bound paclitaxel or infusional 5-fluorouracil, leucovorin/folinic acid, irinotecan, and oxaliplatin (FOLFIRINOX) are commonly used. However, these regimens cause severe cytotoxic and/or gastro-intestinal adverse events and are recommended for the patients with good performance status (PS). Since avadomide has been shown to be effective in patients with severe co-morbidities [29], it can be used as a therapeutic option in patients even with poor PS. In addition, the drug may be used for biliary tract cancer (BTC) because it showed inhibitory activity for cancer growth using 5 BTC cells (Online resource 1, Supplementary Fig. S2).

In addition to first-line chemotherapy investigation, we evaluated efficacy of avadomide in gemcitabine-resistant cells. We showed that avadomide monotherapy exhibited anti-tumor effects on GR PDAC clones by inhibiting the NF-κB process and G1 phase cell cycle arrest. The outcomes were further confirmed using another cancer cell line, CCLP-1 (Online resource 1, Supplementary Fig. S2). Avadomide was sensitive to two different cancer cells that were resistant for gemcitabine; therefore, the former would also be potentially useful as second-line chemotherapy. Currently, the recommended second-line chemotherapy for patients who have gemcitabine resistance is liposomal irinotecan (nal-IRI) combined with 5-FU + LV. Avadomide could thus be a new candidate for second-line chemotherapy.

Avadomide showed growth inhibitory effect regardless of gemcitabine-resistance; however, the target mechanism was unknown. Because avadomide works as a co-factor for proteasome degradation, we initially investigated its action in apoptosis, cell cycles, and association to NF-κB.

Modulation of NF-κB activity and cancer cell progression are regarded as potential targets for the treatment of human malignancies. The NF-κB pathway plays a significant role in regulating cell proliferation, apoptosis, and metastasis, and its over-activation has been observed in nearly all solid tumor cells [30]. Although the activity of NF-κB pathways has been shown to induce gemcitabine resistance in multiple ways, the mechanism of gemcitabine resistance in PDAC remains unclear [6]. In our study, avadomide inhibited pIκBα expression associated with the NF-κB pathway and enhanced cleaved caspase 3 expression in MiaPaCa2-GRs. This suggests that the death receptor signaling may be involved in the anti-tumor effects of avadomide in GR cells.

Moreover, some data have shown that inhibition of the NF-κB pathway suppresses cell cycle progression and chemoresistance by reducing the concentration of cyclins A, B, and D1, and CDKs 4 and 6 [31, 32]. In our study, cyclin kinase inhibitor (CKI), p18INK4C, p21 Waf1/Cip1, and p27Kip1 expressions were elevated, and those of cyclin D1, CDK4, and CDK6 were suppressed after avadomide treatment.

Binding of thalidomide and its analogues, including avadomide, to cereblon, a substrate receptor of the Cullin 4 RING E3 ubiquitin ligase complex, resulted in proteasomal degradation of Ikaros [14]. In multiple myeloma cell lines, the degradation of Ikaros resulted in reduced c-Myc protein levels, resulting in decreased proliferative capacity [33]. In this study, avadomide caused Ikaros degradation both in parent cells and GR clones.

In the in vivo experiment, a xenograft mouse model demonstrated the safety and efficacy of avadomide treatment. Although the viable area in the tissue stained with H&E was observed to be significantly narrow in the avadomide therapy group, notably in the MiaPaCa2-GRs, there was no significant difference in tumor weight. Thus, avadomide was shown to change chemosensitivity through various mechanisms, especially in MiaPaCa2-GRs, and might improve progression-free survival after the incidence of gemcitabine resistance.

In conclusion, the current study demonstrated that avadomide induced apoptosis via NF-κB process inhibition and G1 phase cell cycle arrest. Avadomide could be a novel option for chemotherapy in PDAC, especially in GR patients.

## Supplementary Information

Below is the link to the electronic supplementary material.

Supplementary file1 (DOCX 107 KB)

Supplementary file2 (TIF 1123 KB)

## Data Availability

The datasets generated during and/or analyzed during the current study are not publicly available but are available from the corresponding author on reasonable request.
