# Supplementary material for: Anti-tumor effect of avadomide in gemcitabine-resistant pancreatic ductal adenocarcinoma
Source: Cancer Chemother Pharmacol. 2023 Jul 26;92(4):303–14. doi: 10.1007/s00280-023-04531-w (PMC10435408; doi:10.1007/s00280-023-04531-w)
Supplement: Supplementary file 1 — Supplementary file1 (DOCX 107 KB) [file 280_2023_4531_MOESM1_ESM.docx]

**Supplementary Figures**

**Anti-tumor effect of avadomide in gemcitabine-resistant pancreatic ductal adenocarcinoma**

**Authors:**

Hidemi Nishi, Kunihito Gotoh, Yoshito Tomimaru, Shogo Kobayashi,

Kazuki Sasaki, Yoshifumi Iwagami, Daisaku Yamada, Hirofumi Akita, Tadafumi Asaoka, Takehiro Noda, Hidenori Takahashi, Masahiro Tanemura, Yuichiro Doki, Hidetoshi Eguchi

**Affiliations:**

Department of Gastroenterological Surgery, Graduate School of Medicine, Osaka University, Suita, Japan

**Corresponding author:**

Shogo Kobayashi

E-mail: [skobayashi@gesurg.med.osaka-u.ac.jp](mailto:heguchi@gesurg.med.osaka-u.ac.jp)


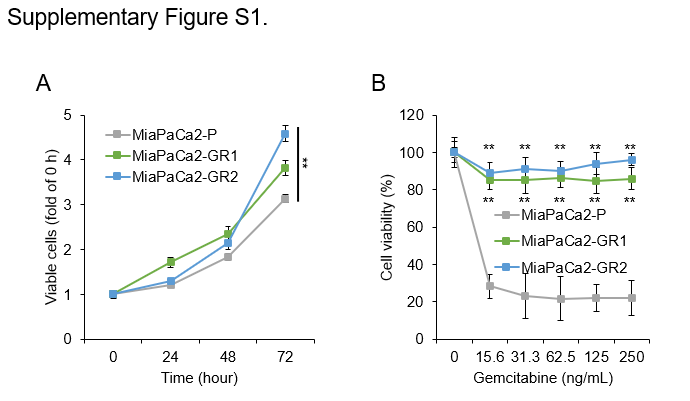


**Supplementary Fig. S1** Characteristics of MiaPaCa2-GR clones and confirmation of chemosensitivity to gemcitabine in MiaPaCa2-GR clones

(**A**) MTT assay showed cell proliferation in MiaPaCa2-GR clones were faster growth curves than in MiaPaCa2-P cells. (**B**) MTT assay confirmed chemoresistance to gemcitabine in MiaPaCa2-GR clones. Data are represented as mean ± S.D. of triplicate independent experiments. **p<0.01, compared with control.


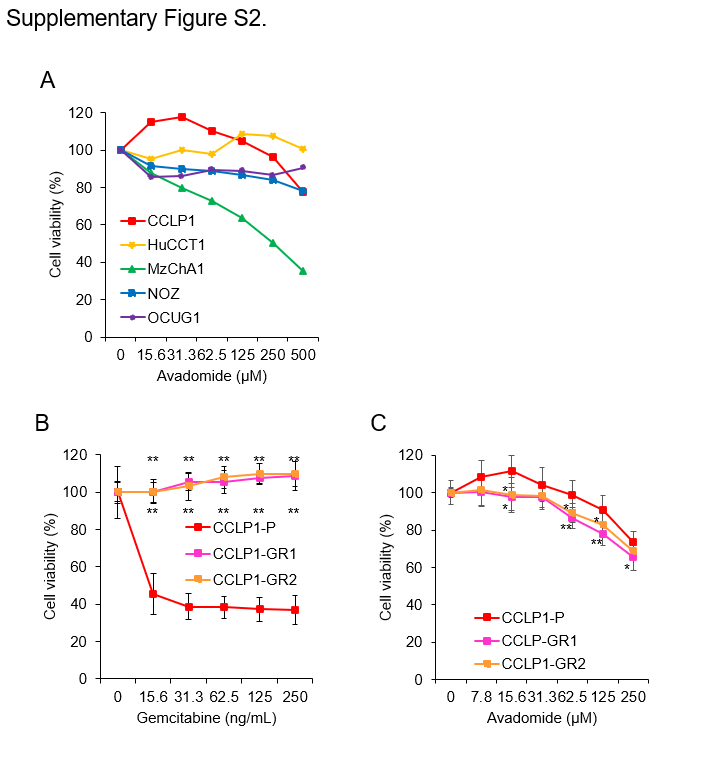


**Supplementary Fig. S2** Chemosensitivity study for BTC cell lines and gemcitabine-resistant BTC clones

(**A**) MTT assay showed the anti-tumor effects of avadomide in several BTC cell lines. (**B**) MTT assay confirmed chemoresistance to gemcitabine in CCLP1-GR clones. (**C**) MTT assay showed the anti-tumor effect of avadomide in CCLP-GR clones. Data are represented as mean ± S.D. of triplicate independent experiments. *p<0.05, **p<0.01, compared with control.

BTC: biliary tract cancer
