# Supplementary figures and images for: Anti-tumor effect of avadomide in gemcitabine-resistant pancreatic ductal adenocarcinoma
Source: Cancer Chemother Pharmacol. 2023 Jul 26;92(4):303–14. doi: 10.1007/s00280-023-04531-w (PMC10435408; doi:10.1007/s00280-023-04531-w)

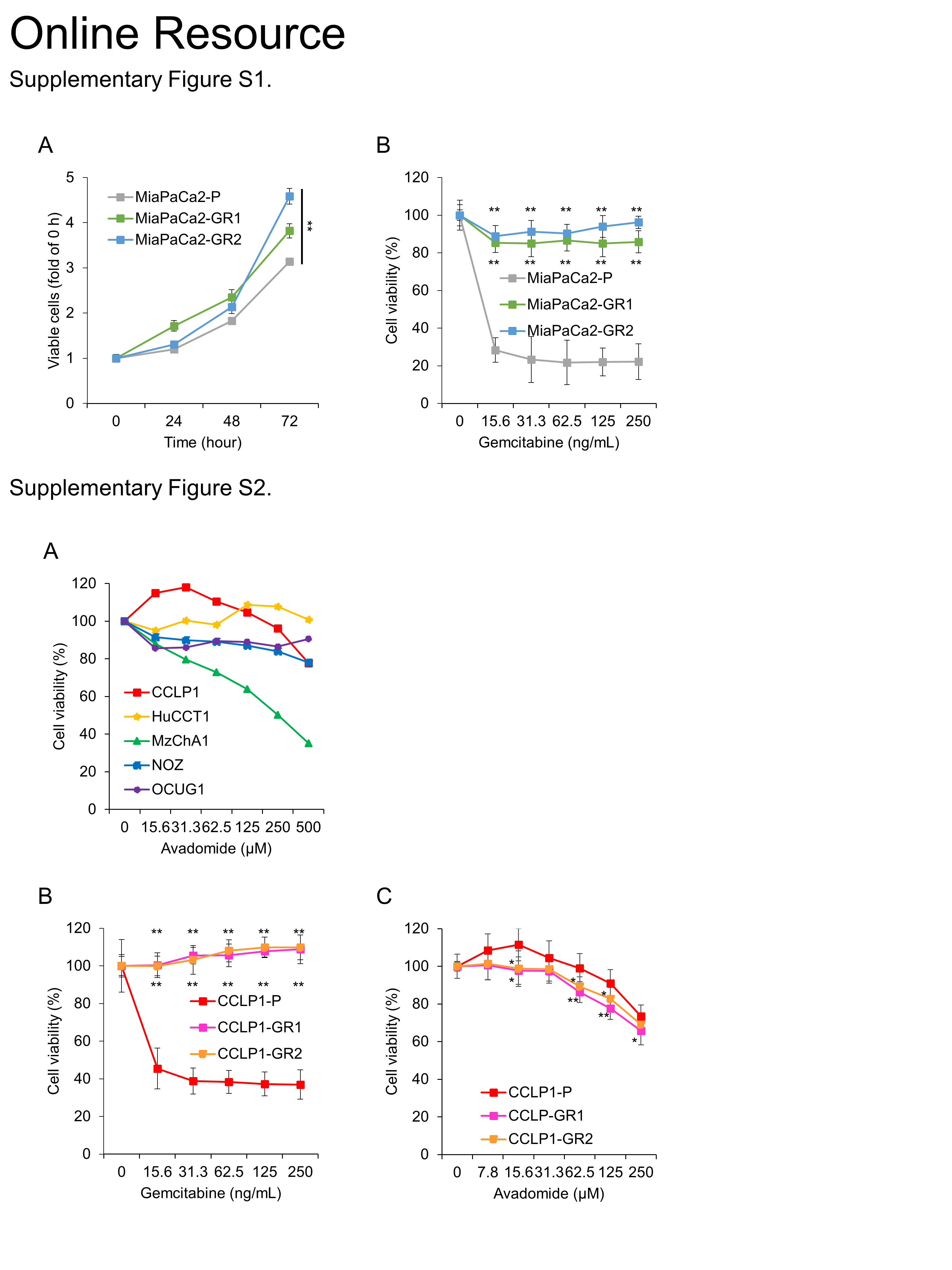

Supplement: Supplementary file 2 — Supplementary file2 (TIF 1123 KB) [file 280_2023_4531_MOESM2_ESM.tif]
